# Editor's Letter-Box

**Published:** 1897-12-04

**Authors:** James Oliver


### Editor's Letter-Box.

[Our Correspondents are reminded that prolixity is a great bar to publication, and that brevity of style and conciseness of statement greatly facilitate early insertion.]

Dr. James Oliver writes: I have read with pleasure and interest your article in The Hospital of November 20th, entitled "Facts and Principles as to Hospital Reform." The facts as therein stated apply even more forcibly to the question of operations, and the difficulty many patients experience in having an operation done by a skilled specialist or general surgeon without having recourse to a charitable institution. Many patients who are admitted into hospital for operation could afford a moderate fee, but the charges made by the home hospitals—£4 4s. a week and upwards, together with the moderate fee asked for the operation—leaves them no alternative but to accept the benefits of a charitable institution. The question of operating at the patient's own home is precluded on account of want of accommodation for one or two nurses, as the case may be, the demands made by nurses, and the fees charged for their services.

